# Nanostructured Lipid Carriers-Hydrogels System for Drug Delivery: Nanohybrid Technology Perspective

**DOI:** 10.3390/molecules27010289

**Published:** 2022-01-04

**Authors:** Sharifah Nurfadhlin Afifah Syed Azhar, Siti Efliza Ashari, Norhazlin Zainuddin, Masriana Hassan

**Affiliations:** 1 Integrated Chemical BioPhysics Research Centre (iCheBP), Faculty of Science, Universiti Putra Malaysia (UPM), Serdang 43400, Selangor, Malaysia; snurfadhlin@gmail.com; 2 Department of Chemistry, Faculty of Science, Universiti Putra Malaysia (UPM), Serdang 43400, Selangor, Malaysia; norhazlin@upm.edu.my; 3 Centre of Foundation Studies for Agricultural Sciences, Universiti Putra Malaysia (UPM), Serdang 43400, Selangor, Malaysia; 4 Department of Pathology, Faculty of Medicine and Health Sciences, Universiti Putra Malaysia (UPM), Serdang 43400, Selangor, Malaysia; masriana@upm.edu.my

**Keywords:** nanostructured lipid carriers, hydrogel, drug delivery, nanohybrid system

## Abstract

Advanced hybrid component development in nanotechnology provides superior functionality in the application of scientific knowledge for the drug delivery industry. The purpose of this paper is to review important nanohybrid perspectives in drug delivery between nanostructured lipid carriers (NLC) and hydrogel systems. The hybrid system may result in the enhancement of each component’s synergistic properties in the mechanical strength of the hydrogel and concomitantly decrease aggregation of the NLC. The significant progress in nanostructured lipid carriers–hydrogels is reviewed here, with an emphasis on their preparation, potential applications, advantages, and underlying issues associated with these exciting materials.

## 1. Introduction

The structural combination of a polymer hydrogel network and a nanoparticle (e.g., metals, nonmetals, metal oxides, and polymeric moieties) possesses the capability of providing superior functionality to the composite material. It has been demonstrated that this combination has practical applications in various fields such as catalysis, electronics, bio-sensing, drug delivery and environmental remediation. Furthermore, the hybridization of the two compounds may enhance the synergistic properties of each component. For instance, the mechanical strength of the hydrogel could simultaneously decrease the nanoparticle’s aggregation, making it more stable and more efficient. As a result of these mutual benefits and the associated potential applications, multidisciplinary research groups have shown a surge of interest in the last decade [1]. Because of their differences in properties when compared to bulk materials, nanoparticles are now widely being used in everyday consumer products and appliances. This trend has sparked public debate about the safety of nanoparticle technology, and regulatory authorities in several countries have stepped in on this issue [2]. The advantages of a nanoscale drug delivery system are numerous and include a targeted delivery method that allows for enhanced drug concentration at the intended place while decreasing systemic exposure to a potentially toxic chemical. In addition, a nanoscale drug delivery system offers a consistent rate of drug administration and an improvement in drug stability owing to drug degradation and loss prevention [3].

Lipidic drug delivery systems have gained attention in recent decades due to their biocompatibility as well as their ability to permeate challenging physiological barriers, particularly the blood–brain barrier (BBB), due to their lipophilicity, even without surface modifications. Additionally, the simplicity of preparation, cost-effectiveness, and practicality of large-scale manufacturing make these delivery methods more appealing [4]. Lipid carriers can be categorized into various types depending on their method of preparation and physicochemical characteristics. They include liposomes, niosomes, solid lipid nanoparticles and nanostructured lipid carriers.

Currently, a variety of nanostructured lipid carriers (NLC) formulations have been approved because they not only demonstrated enhanced loading efficiencies but also improved stability and prevented drug ejection during storage. Improved loading efficiency is attained in the case of hydrophobic drugs due to the natural tendency of certain drugs to dissolve in liquid better than solid lipids, and it has already been established that increased drug solubility leads to higher loading efficiencies [5]. In the case of hydrophilic drugs, on the other hand, a lipid conjugation strategy is used, in which the drug’s functional group (e.g., amine group) is conjugated with the functional group (e.g., carboxylic acid group) of lipids like oleic acid by carbodiimide or another sort of chemistry [6].

Most of the lipids used for the manufacture of these particles are of the Generally Recognized as Safe (GRAS) category and have low toxicity concerns. However, the development of NLC systems may include screening of various components such types of solid lipids, types of liquid lipids, ratios of solid to liquid lipids and types of surfactants. Due to the availability of a wide range of lipids, oils and surfactants, screening of these components is a cumbersome process. Apart from development, NLC are also associated with some serious quality issues such as polymorphic changes in lipids, gelation, presence of supercooled melts, presence of different colloidal species and sterilization stability [7]. The challenges and concerns of applying NLC in drug delivery could be overcome by incorporating them into hydrogels, resulting in lower risks to human health and maximizing the sustained release of the drug. The preparation, advantages and limitations of a nanohybrid system between nanostructured lipid carriers and hydrogels is further discussed in this work.

## 2. Nanostructured Lipid Carriers

**Figure 1:**
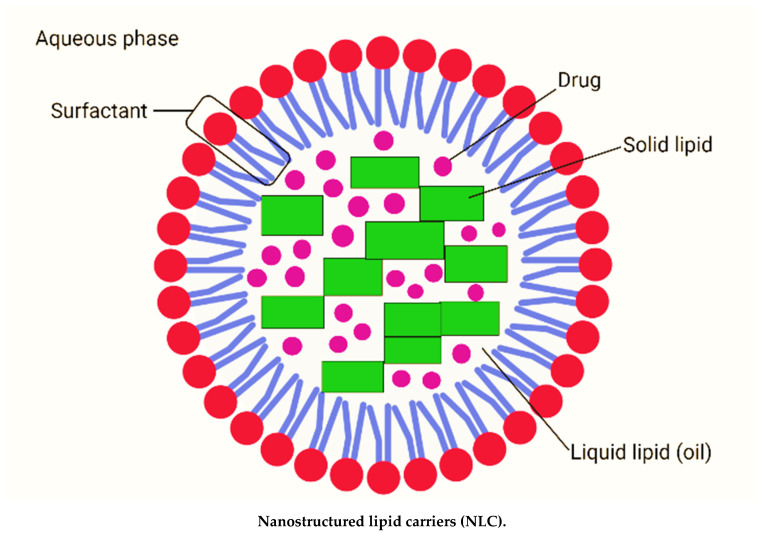
Composition of nanostructured lipid carriers (NLC) consisting of solid lipid, liquid lipid (oil), drug, surfactant and aqueous phase (created with biorender.com (accessed on 2 September 2021)).

Nanostructured lipid carriers (NLC) are lipid nanoparticles of the second generation made up of solid lipid matrices mixed with liquid lipids (oils) [8]. NLC are typically between 200 and 400 nm in size. The different nanosizes of NLC are determined by different preparation techniques. Long-term flocculation and creaming have been demonstrated to make the upper nanosize range > 700 nm less stable. Producing sizes smaller than 200 nm necessitates higher surfactant concentrations, which are often undesirable in formulations. However, NLC with a size of 100 nm frequently have problems because they recrystallize. However, for some applications, NLC with a size of 100 nm are of particular interest due to their superior ability to penetration into the skin. To address this, Baiseng et al. (2013) developed a method for matching the lipid phase’s required hydrophilic–lipophilic balance (HLB). NLC can strongly immobilize drugs and prevent particles from coalescing due to their solid matrix [9,10]. Consequently, the mobility of the incorporated drug molecules is greatly reduced in the solid phase. In addition, the liquid oil droplets in the solid matrix increase the drug loading capacity, while the less ordered lipid matrix allows better drug accommodation [11]. Figure 1 depicts the composition of nanostructured lipid carriers (NLC).

### 2.1. Preparation of Nanostructured Lipid Carriers (NLC)

In order to prepare of NLC, various formulation approaches exist, which include high-pressure homogenization (HPH) [12,13,14], solvent emulsification-evaporation [15], phase inversion [16], high speed homogenization and/or ultrasonication [17], and solvent injection [18]. The main advantages and limitations of these preparation techniques have been discussed in Table 1.

#### 2.1.1. High-Pressure Homogenization (HPH)

HPH technology has proven itself as a reliable and effective method for producing lipid nanoparticles. This approach may also be employed for large-scale manufacturing, unlike previous ones. There were two types of homogenization methods developed: hot and cold. In both methods, the pharmaceutical ingredient is dissolved or disseminated in melted lipid prior to HPH. The fluid in the homogenizer is moved by high pressure (100–2000 bar). The average particle size is sub-micron. Homogenization offers various advantages, including large-scale manufacturing, the lack of organic solvent, increased product stability, and enhanced drug loading, but it is difficult to use due to high pressure and temperature conditions [19].

#### 2.1.2. Solvent Emulsification–Evaporation

The lipid is dissolved in a water-insoluble organic solvent in this procedure. Following that, an emulsion in an aqueous phase with surfactant is created. Evaporation under lower pressure is used to remove the solvent from the emulsion. Evaporation causes nanoparticles to disperse in the aqueous phase (using lipid precipitation process in the aqueous phase). This approach, unlike cold homogenization, will not be subjected to thermal stress; nonetheless, the organic solvent utilized in this process is a drawback. The particle size varies depending on the solid lipid and surfactant [15].

#### 2.1.3. Phase Inversion

Transformation of an o/w type to a w/o type of emulsion is termed “phase inversion”. It can be induced by changing the temperature, and the temperature at which the inversion occurs is referred to as the PIT. This technique mainly depends on the change in the properties of polyoxyethylated surfactants at different temperatures. The hydrophilic–lipophilic balance (HLB) value of surfactants defined by Griffin is valid at 25 °C. At this temperature, the hydrophilic parts of the surface-active compounds are hydrated to a certain extent. Moreover, the dehydration of the ethoxy groups occurs when an increase in temperature. Thus, the lipophilicity of the molecules of the surface-active compounds rises with the decrease in HLB value. At a certain point, the surface-active compounds with affinity to the aqueous and lipid phase are equal—this temperature is called the phase inversion temperature. In this method, lipid, drug, water and surfactant are mixed together by magnetic stirring, and three heating and cooling cycles are performed. The mix is then diluted with cold water causing phase inversion of the emulsion and breaking, which results in the NLC [16].

#### 2.1.4. High Speed Homogenization and/or Ultrasonication

Lipid nanoparticle dispersions are obtained by dispersing the melted lipid in the warm aqueous phase containing surfactants by high sheer homogenization followed by ultrasonication. This method primarily involves heating of a solid lipid to approximately 5–10 °C above its melting point. The lipid melt is dispersed in an aqueous surfactant solution at the same temperature under high-speed stirring to form an emulsion. Subsequent sonication reduces the droplet size of the emulsion. Gradual cooling of the warm emulsion below the crystallization temperature of the lipid yields a lipid nanoparticle dispersion. Concentrated lipid nanoparticle dispersions can be obtained by ultracentrifugation [15,17].

#### 2.1.5. Solvent Injection/Displacement

In the solvent injection method, lipids are dissolved in a water-miscible solvent like acetone, isopropanol, and methanol water-miscible solvent mixture and quickly injected into an aqueous solution of surfactants through an injection needle. The gradual solvent diffusion out of lipid-solvent droplets into water causes reduction of droplet size and simultaneously increases lipid concentration. In addition, the diffusion of pure solvent from the lipid-solvent droplet causes local variations in the interfacial tension at droplet surface, inducing reduction of particle size of NLC [18].

### 2.2. Advantages and Limitations of Nanostructured Lipid Carriers

In terms of drug release, stability, and dispersion in long-term storage, NLC outperform the first generation of lipid nanoparticles, which are solid lipid nanoparticles (SLN) [7]. The unique preparation of NLC using a different blending of solid lipids with liquid lipids (oils) results in a lipid particle-matrix with a lower melting point than the original solid lipid. Nevertheless, this matrix remains solid at body temperature [11]. The solid lipid undergoes polymorphic changes in the SLN system, such as recrystallization in a low melting, less stable modification and change to a more stable modification over time. The drug can be expelled due to changes in the crystalline structure, resulting in the precipitation of large drug crystals in the water phase.

To address this issue, adding oil to a lipid can prevent the lipid from re-crystallizing in a less stable form, as demonstrated in a few studies [20,21]. As a result, no changes in modification occur in NLC over time, and thus, no drug expulsion is obtained [22]. As no sophisticated equipment is required, the preparation of NLC is significantly less expensive and cost effective. Furthermore, NLC have a high drug loading and drug release that the particle-matrix material could modulate. As a result, the release of the drug is controlled and is not limited or solely determined by size. NLC have a clear benefit in terms of low toxicity value for their market, as they could be made from orally accepted lipids and surfactants, allowing for concept development. Lipid nanoparticles could also be useful for intraocular delivery via injection. Previous research has shown that NLC particles are biodegradable and have excellent tissue tolerability when injected into chickens [23].

Furthermore, autoclaving can be used to sterilize lipid nanoparticle suspensions. During the process, the particles melt and re-crystallize. As a result, they meet the essential requirements for sterile, biodegradable, and tissue-tolerable intraocular formulations. However, this technique has some limitations. NLC require a strong dilution of the particle dispersion that often yields less than 1% particles and the need to remove organic solvent residues. The main barrier to industrial use is the possibility of obtaining very low concentrated particle suspensions. In addition, many final products, such as tablets, require excessive water to be removed [24].

## 3. Hydrogels

Hydrogels are three-dimensional cross-linked polymer networks that absorb a large amount of water when placed in an aqueous solution [25]. This unique feature makes them soft and wet materials with both solids and liquids characteristics, such as a large amount of free water and potentially soluble molecules that can diffuse in and out of the gels. These polymers have good swelling properties in addition to non-toxicity, biocompatibility, and biodegradability. Due to their three-dimensional cross-linked hydrophilic networks, which can hold large amounts of water (typically in the range of 30–90%) [26]. Because of their wide variety of biocompatible matrices and biologically active materials such as chitosan, cellulose, starch, alginate, and neutralized polyacrylic acid (PAA), hydrogel-based wound dressings are currently of great interest to scientists all over the world [27,28,29].

Hydrogels can be made from two types of polymers: synthetic polymers or natural polymers. Synthetic polymers are generally water-soluble materials that dissolve, disperse, or swell in water, changing the physical properties of aqueous systems by gelation, thickening, or emulsification/stabilization. These polymers are often composed of repeating units or blocks of units containing hydrophilic groups (nonionic, anionic, cationic, or amphoteric) as substituents or incorporated into the polymer chains [30]. PEG (poly (ethylene glycol)) is a valuable synthetic polymer with a high solubility in organic solvents. Furthermore, PEG is appropriate for biological applications because of its aqueous solubility and low intrinsic toxicity. When coupled with hydrophobic medicines or carriers, PEG’s strong hydrophilic nature increases their solubility. It improves the physical and chemical stability of pharmaceuticals and inhibits drug aggregation in vivo and during storage [31].

Moreover, polyacrylic acid (PAA) is a biodegradable, water-soluble polymer that has a variety of commercial uses, including super adsorption and water treatment. PAA is remarkable in that it occurs as a liquid at pH 5 and as a gel at pH 7. Cation permeation into the gelled polymer turns it back to a liquid. It is suitable as a medication delivery vehicle for ocular administration of ribozymes to the corneal epithelium [32]. PAA-based polymers are mostly employed in oral and mucosal contact applications such controlled release tablets, oral solutions, and bio-adhesives. They are also employed in low viscosity systems for topical treatments as thickening, suspending, and emulsion stabilizing agents [33].

The structure of polyvinyl alcohol (PVA) includes a hydroxyl group. It is produced by polymerizing vinyl acetate to polyvinyl acetate (PVAc), which is subsequently hydrolyzed to produce PVA [34]. PVA is soluble in water, Dimethyl Sulfoxide (DMSO), Ethylene Glycol (EG), and N-Methyl Pyrrolidone (NMP) [32]. The most significant solvent for PVA is water. Degree of polymerization (DP), hydrolysis, and solution temperature all influence PVA solubility in water. Any change in these three parameters influences the degree and nature of hydrogen bonding in aqueous solutions and hence PVA solubility. Temperature, concentration, percent hydrolysis, and molecular weight of PVA affect its solubility, viscosity, and surface tension [35]. Because PVA hydrogels are non-toxic, non-carcinogenic, and bioadhesive, they have been employed in a variety of biological and pharmacological applications [36]. PVA also has a high degree of swelling in water (or biological fluids) as well as a rubbery and elastic quality, and thus, it closely resembles real tissue and is easily absorbed by the body. PVA gels have been applied in contact lenses, prosthetic heart linings, and drug delivery systems [37].

Natural polymers, on the other hand, are typically made up of protein and extracellular matrix components, as well as natural material derivatives. Chitin and chitosan are widely employed in a variety of sectors, including food processing, waste management, medicine, biotechnology, and pharmaceuticals. Since it is biodegradable, biocompatible, and less toxic, chitosan has been extensively utilized as a formulation excipient in pharmaceutical applications. It has been utilized as a mucoadhesive, an oral absorption enhancer, and in the delivery of protein and genes [38]. However, chitin and chitosan have the disadvantage of being difficult to dissolve in water and at a neutral pH. As a result, many researchers have used chemical modification to create water soluble chitosan and chitosan derivatives. These chemical changes cause hydrophilic chitin or chitosan to develop, which has a higher affinity for water or organic solvents [39]. Chemical modification has overcome chitosan and chitin’s limited solubility. For example, carboxymethylation of chitosan results in the creation of N-carboxymethylchitosan (N-CMC), which is soluble in a wide pH range. Due to their affinity towards metal ions, chitin and chitosan derivatives are also commonly used in the treatment of industrial wastewater. N-CMC has been widely employed in the pharmaceutical industry to provide controlled release of pharmaceuticals, orthopedic devices, and connective tissue [40].

**Figure 2:**
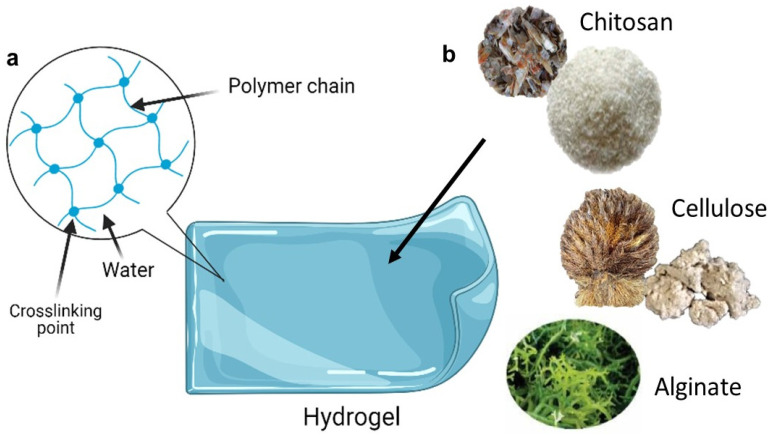
Schematic diagram of hydrogels: (**a**) structural composition of hydrogels; (**b**) examples of natural polymers networks (clockwise from top right) chitosan from shrimp waste, cellulose from oil palm biomass, and alginate from brown algae (created and modified with biorender.com (accessed on 2 October 2021) and adapted from [43,44,45,46]).

Chondrus crispus, Eucheuma cottonii, and Eucheuma spinosum are the primary sources of carrageenan. It is a natural component derived from specific species of red seaweed (Rhodophyceae). It is composed of repeating galactose units and 3,6-anhydrogalactose (3,6-AG), both sulfated and non-sulfated, linked by alternating (1-)-and (1-4)-glycosidic connections. In hard and soft gel capsules, carrageenan is thought to be a good alternative for gelatin (an animal-based substance). The inclusion in a glycerin-water combination hides the chalkiness of ant-acid gels. It can be utilized in both topical and suppository formulations. Carrageenan is utilized in hand lotions and shampoos as a thickening agent, encouraging healthy skin and hair. Carrageenan possesses distinct features such as viscosity, continuous phase gel formation, and specialized interactions with abrasives [41,42]. Figure 2 shows the schematic diagram of hydrogel.

### 3.1. Preparation of Hydrogel

Hydrogels can be made from almost any water-soluble polymer, and they can have a wide range of chemical compositions and bulk physical properties. Furthermore, hydrogels can be manufactured in a variety of physical forms, such as slabs, microparticles, nanoparticles, coatings, and films. As a result, hydrogels are commonly used in clinical practice and experimental medicine for a wide range of applications, including tissue engineering and regenerative medicine [47], diagnostics [48], cellular immobilization [49], separation of biomolecules or cells [50], and barrier materials to regulate biological adhesions [51]. Hydrogels’ distinct physical properties have sparked particular interest in their use in drug delivery applications. Controlling the density of cross-links in the gel matrix and the affinity of the hydrogels for the aqueous environment in which they are swollen allows them to easily tune their highly porous structure.

Their porosity also allows drugs to be loaded into the gel matrix and be released at a rate determined by the diffusion coefficient of the small molecule or macromolecule through the gel network. Indeed, the advantages of hydrogels for drug delivery may be primarily pharmacokinetic, in that a depot formulation is created from which drugs slowly elute, maintaining a high local concentration of drug in the surrounding tissues for an extended period of time, though they can also be used for systemic delivery. Hydrogels are also generally biocompatible, as evidenced by their effective use in the peritoneum and other sites in in vivo [52]. The high water content of hydrogels and their physicochemical similarity to the native extracellular matrix, both compositionally (particularly in the case of carbohydrate-based hydrogels) and mechanically, promote biocompatibility. To design biodegradability or dissolution into hydrogels, enzymatic, hydrolytic, or environmental (e.g., pH, temperature, or electric field) pathways can be used. Degradation, however, is not always desirable depending on the time scale and location of the drug delivery device. Hydrogels are also malleable, which allows them to conform to the shape of the surface to which they are applied [53].

The preparation of hydrogels can be divided into physical cross-linking and chemical cross-linking. The effect of cross-linking determines the physical and chemical properties and functions of the hydrogel. Crosslinking is the most critical step in preparing hydrogel to hold the 3D structure and improve hydrogels’ physical and mechanical properties [54]. Table 2 summarizes the preparation method of hydrogel with its advantages and limitations.

#### 3.1.1. Physical Cross-Linking

In the preparation of hydrogel, physical crosslinking has been an interesting technique due to the type of crosslinking agents used. In drug delivery systems, the physical crosslinking preparation was usually selected since it does not interrupt the living organism and improves the hydrogel structures [55]. There are various techniques used in physical cross-linking as describe below.

##### Ionic Interaction

Metal-ligand interaction, which is based on the dynamic interaction of oppositely charged groups, is an efficient method of carrying out ionic interactions. Ionic interaction produces hydrogels with high ionic conductivity, fatigue resistance, environmental response, and self-healing properties. However, the poor mechanical properties and complex preparation process of hydrogels formed by ionic interactions continue to be the main issues preventing their further application [56]. At the moment, an increasing number of researchers are concentrating on developing new hydrogels to address these issues. For example, negatively charged monomer acrylic acid (AAC) and positively charged 2-hydroxypropyl trimethylammonium chloride chitosan (HACC) interact to form a high-density dynamic ionic bond of the PAAC/HACC hydrogel’s compact structure. The structure gives the hydrogels good mechanical properties, ionic conductivity, and self-healing properties.

The ionic conductivity is sufficient to transfer bioelectrical signals and electrical stimulation for cell proliferation and differentiation to the human body [57]. Liu et al. 2018 used a dynamic ionic bond cross-linking to create CNF/G/Ag0.5 interpenetrating polymer network hydrogels (IPN). The hydrogels clung to the wound’s surface, causing platelet aggregation. Gelatine can promote erythropoiesis and increase the number of platelets and white blood cells to prevent bleeding, and modified cross-linked hydrogels can increase water absorption efficiency and decrease water vapour diffusion, resulting in a decrease in water vapor transmission rate (WVTR) to maintain an appropriate balance of fluids on the wound bed [58].

##### Hydrogen Bond

The utilization of hydrogen bonds is frequently required, and hydrogen bonding can considerably increase hydrogel self-repair and self-recovery capacities [59]. However, because hydrogen bonds are generally fragile in aquatic environments, hydrogels with poor usage rates are common. Researchers are now working to increase the impact of hydrogen bonding by creating double network hydrogels or IPN hydrogels [60]. On the basis of numerous hydrogen bond interactions, Bi et al. created physically cross-linked chitosan-polyvinyl alcohol double network hydrogels. Hydrogels can be spontaneously recreated after being destroyed because the hydrogen bond interaction is a dynamic interaction. Physical cross-linking hydrogels are also biodegradable and cell compatible [61]. Zhao et al. promoted sodium alginate (SA) self-assembly in the polyacrylamide (PAM) porous matrix via hydrogen bonding. Polyacrylamide/sodium alginate (PAM-SA) semi-interpenetrating polymer network hydrogels exhibit strong mechanical characteristics as well as high self-healing efficiency at room temperature due to hydrogen bond interaction. This feature can help the hydrogels last longer in various applications, especially under extreme conditions [62].

##### Freeze-Thawing

One of the most frequent techniques of physical crosslinking is the freeze–thaw procedure. The generated ice crystals arrange the polymer chains around themselves during the freezing phase of the cycle. The ice crystals melt to produce a microporous structure throughout the cycle’s thawing process [63]. For varied pore sizes, mechanical strengths, morphology, or other features, the duration, temperature, number of cycles, and amount of polymer components can be adjusted during the freeze-thaw process. The soft, flexible, and changeable porosity of freeze-thaw hydrogels can mimic extracellular matrix (ECM), and stem cells put on it can sense and respond to dynamic changes in ECM stiffness and respond and move in a directed way, which is critical for recruiting cells for wound healing [64].

##### Chemical Cross-Linking

Chemical cross-linking is currently used to make the majority of hydrogel polymers. Chemically cross-linked hydrogels are frequently more stable and have better mechanical characteristics [65]. Enzymatic reaction, free radical polymerization reaction and conjugation process the common techniques.

##### Enzymatic Reaction

Enzymes such as transglutaminase, tyrosinase, urease, and horseradish peroxidase (HRP) catalyze the cross-linking of natural polymers [66]. Enzymatic reactions take place at mild circumstances, preventing biological activity loss and quick gelation, and no toxic compounds are formed. The application of enzymatic fast gelation to generate antibacterial hydrogels is currently promising [67]. Scientists are increasingly interested in 3D cell culture of hydrogels; building a hydrogel network with reversible stiffening/softening capability is critical, and enzymatic reactions can provide substrate specificity and mild/predictable reaction kinetics [68]. Transglutaminase can be used to induce the covalent connection of HA and PEG macromers, and in situ hyaluronic acid hydrogels can selectively modulate cell phenotype by altering their own mechanical and biochemical characteristics [69].

##### Free Radical Polymerization

Heating, UV radiation, high energy radiation, electrolysis, and plasma initiation are all created by free radicals [70]. Thermally initiated polymerization and light-initiated polymerization both use unsaturated functional groups or photosensitive functional groups to undergo free radical polymerization or cross-linking under the action of heat or light to produce covalent bonds [71]. The majority of the hydrogels produced by thermally induced cross-linking processes are suitable for deep wound healing, and their structure is stable and highly controlled. The precursor having the photosensitive functional group can be directly polymerized under UV radiation in the photo-initiated polymerization process, while the precursor containing the double bond functional group can be polymerized under UV radiation by adding a photo initiator [72].

##### Conjugation Reaction

The Michael addition reaction, the Schiff’s base reaction, and the Diels–Alder addition reaction are all conjugation reactions that may be carried out under very moderate circumstances [73]. In the conjugate reaction, the Schiff’s base reaction (condensation of amine and active carbonyl group) is a simple green approach [74]. Many polysaccharide molecules, such as alginate, starch, hyaluronic acid, and cellulose, include adjacent hydroxyl groups that may be oxidized by periodate to create hydrogels via Schiff’s base reactions [75]. Using oxidized hydroxyethyl starch (O-HES) and modified carboxymethyl chitosan (M-CMCS) as raw materials, the Schiff’s base reaction was used to create an injectable in situ hydrogel with excellent self-recovery, biocompatibility, biodegradability, and transparency that can be injected into irregular-shaped skin defects and formed in situ to shape the contour of various dimensions. The excellent compliance made hydrogels easy to adapt to the wound under different conditions of skin movement, and full-thickness skin defects treated with M-CMCS/O-HES hydrogels demonstrated this promising therapeutic strategy for wound healing [76].

**Table 2:** Preparation method of hydrogel with its advantages and limitations.

Type of Cross-Linking	Method	Advantages	Limitations	References
Physical	Ionic interaction	High ionic conductivity, endurance strength and self-healing properties.	Poor mechanical properties and complex preparation process of hydrogels	[56]
	Hydrogen bond	Increase hydrogel self-repair and self-recovery capacities. Strong mechanical characteristics	Fragile in aquatic environments and poor usage rates	[59]
	Freeze -thawing	Soft, flexible, and changeable porosity	Opaque appearance and the limited swelling capacity and thermal stability.	[64]
Chemical	Enzymatic reaction	High biological activity, quick gelation, and non- toxic.	Most expensive crosslinker	[67]
	Free radical polymerization	Structure is highly stable and controlled.	Difficulty of preparing well-defined copolymers or polymers with a predetermined functionality.	[72]
	Conjugation reaction	Excellent self-recovery, biocompatibility and biodegradability	Use harsh chemicals	[76]

### 3.2. Advantages and Limitations of Hydrogel

According to the previously cited authors, hydrogel has been used as a technology to deliver products suitable for medical use, particularly in wound management. Hydrogels generate a moist environment and good fluid absorbance conducive to a successful wound healing process [77,78]. Furthermore, hydrogel pharmaceutical preparations are preferred over creams due to their higher water content in the form of gels, which aids in pain reduction when applied, particularly to mucous membranes or injured or burned skin [79]. Hydrogels have advantages over ointments when used for dermatological purposes, such as being emollient, thixotropic, and greaseless. Furthermore, researchers discovered that polydopamine loaded with nanocellulose hydrogel has promising wound healing and repair results. When compared to the blank and nanocellulose hydrogel samples, the in vivo skin defects experiments revealed that the composite hydrogel had a synergistic effect on wound healing. Notably, the wound took 10 days to heal, while the size of the wound circle was reduced after 15 days. This phenomenon could be attributed to the strong adhesion of composite hydrogels to wet mucosal and tissue surfaces due to the strong adhesion ability of catechol groups of polydopamine. The catechol groups on polydopamine could act as binding sites for adherent tissues [80].

Moreover, Nakasone and Kobayashi (2016) generated interest in developing cellulose hydrogels by using sugarcane bagasse waste as a cellulose resource to prepare transparent and flexible cellulose hydrogel films. According to the findings, the cellulose hydrogel film with a trace of lignin demonstrated acceptable cytocompatibility on NIH 3T3 mouse embryonic fibroblast cells in terms of protein adsorption, cell density, and proliferation rate [81]. As a result, cellulose hydrogel films made from waste biomass are cytocompatible with fibroblasts, important cells in wound healing. As for skin regeneration, it is well known that a hydrogel scaffold can provide a moist covering for skin repairing while also protecting the wound from infection [82].

Despite their numerous advantages, hydrogels have several drawbacks. For example, the low tensile strength of hydrogels’ limits their use in load-bearing applications, resulting in premature dissolution or flow away from a locally targeted site. In many common drug delivery applications, such as subcutaneous injection, this limitation may not be significant. The amount and homogeneity of drug loading into hydrogels may be limited, especially for hydrophobic drugs. The high water content and large pore sizes of most hydrogels’ result in relatively rapid drug release, which last from a few hours to a few days. The ease of application can also be a problem; while some hydrogels are sufficiently deformable to be injectable, many are not and must be implanted surgically. Each of these issues significantly limits the clinical application of hydrogel-based drug delivery therapies [83].

## 4. Nanohybrid System: Nanostructured Lipid Carrier-Hydrogel

**Figure 3:**
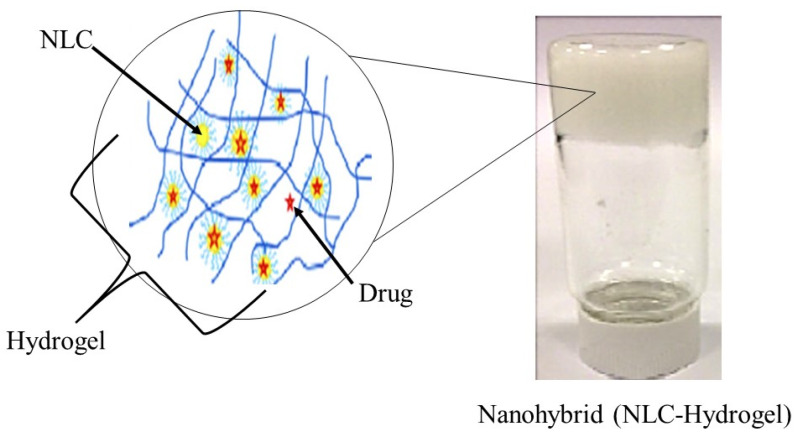
Structural view of nanohybrid system (NLC-Hydrogel) (created and modified with biorender.com (accessed on 2 October 2021) and adapted from [89]).

Typically, a semi-solid vehicle is required to disperse colloidal carrier formulations to develop suitable formulations composed of nanostructured lipid carriers (NLC) for topical, dermal, and transdermal administration. Recently, NLC hydrogel formulations have been described as one of the possible semi-solid systems for drug administration via topical, dermal, and transdermal routes [84]. The process of material hybridization is an ancient practice, as reported by the ancient Egyptians [85]. These advanced hybrid materials combine each excipient’s structural, physicochemical, mechanical, and therapeutic properties in a single final form in the drug delivery system field. Hybridization allows the creation of smart formulations or pharmaceutical forms capable of interacting specifically with biological barriers such as mucosal tissues and skin [86]. Besides that, previous studies of lipid-biopolymer hydrogels for the topical sustained release of ketoprofen [87], ofloxacin [88] and resina draconis [89] exhibited optimized properties in comparison to their lipid-related drug delivery system. Figure 3 shows a structural view of a nanohybrid system.

### 4.1. Conceptualization of NLC-Hydrogel

**Figure 4:**
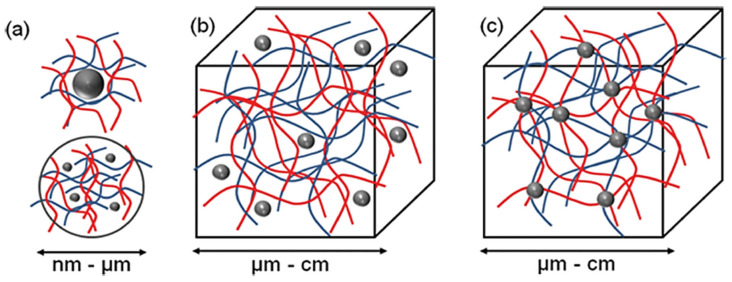
The concept for a combination of NLC and hydrogel to form new functional materials. Three different structural designs exist: (**a**) nano-sized hydrogel particles stabilizing inorganic or polymer NLC (**b**) NLC non-covalently immobilized in a hydrogel matrix and (**c**) NLC covalently immobilized in hydrogel matrix (Adapted from [1]).

Three different supramolecular NLC-hydrogel designs can be proposed: (a) nano-hydrogels stabilizing single or multiple NLC, (b) NLC non-covalently immobilized in a hydrogel matrix and (c) NLC covalently immobilized in a hydrogel matrix (Figure 4).

According to Figure 4, the most basic method for forming an NLC-hydrogel composite is gelation of a suspension of pre-formed nanoparticles in a hydrogel-forming monomer solution. This method was used to create optically responsive optomechanical nanoparticle-hydrogel composites [90]. However, there are some downsides to this method, including the leaching of nanoparticles from the hydrogel matrix if the cross-link density is low [91]. Additionally, crosslinking groups present on the nanoparticle surface are used in the development of nanoparticle-hydrogels. The flexibility of nanoparticles as cross-linkers to form multiple bonds within gel networks (multivalency), as opposed to the two covalent bonds of a traditional hydrogel formation reaction, is a significant advantage. Furthermore, the incorporation of nanoparticles into hydrogels was shown to result in increased interfacial binding between the network and the nanoparticles, resulting in increased stiffness as well as excellent energy dissipation capability with orders of magnitude improvement in fracture resistance under compressive loading [92]. The novel combination of these two very different types of materials is expected to produce not only structural diversity but also a slew of property enhancements. Recent evidence, for example, by Liu et al. (2014), found that a silica nanoparticle-hydrogel composite made of silica nanoparticles and modified polyethylene glycol demonstrated notable improvement in tissue adhesiveness, mechanical stiffness, and bioactivity when compared to a hydrogel without nanoparticles [93]. In addition, when gold nanoparticles were immobilized in Poly-N-isopropyl amide hydrogels, significant changes in mechanical property and thermal response were also observed [94].

Tomsic et al. (2009) and Guillot et al. (2010) were the first to demonstrate the reversible incorporation of nanostructured lipid particles in hydrogels (2009) [95]. Kulkarni et al. (2011) extended the concept to the formation of dry films. Rehydration and re-dissolution of gel films could result in the recovery of nanostructured lipid particles. The size and nanostructure of lipid particles were preserved as a result of the aforementioned hysteresis [96]. Kulkarni et al. (2015) conducted one study that used a nanohybrid system of nanostructured lipid particles and polysaccharide-based hydrogel for controlled therapeutic applications for drug delivery. The nanostructured lipid particles were made by kinetically stabilizing self-assembled lipid nanostructures, and the hydrogel was made by dissolving kappa-carrageenan (KC) in water. The drug was incorporated into both native and lipid particle-loaded hydrogels, which formed thin films upon dehydration and demonstrated improved instability, as well as the ability to release more drug in an efficient manner [90].

### 4.2. Why Nanohybrids?

Scientists have spent decades studying nanohybrids between NLC and hydrogels. Hydrogels are now used in a wide range of biomedical applications, including drug delivery [97], wound dressing [98], and antimicrobial applications [99]. Most of these applications necessitate the use of multifunctional hydrogels and dynamic interactions with the cellular microenvironment [100]. However, one of their major limitations is their low mechanical strength, which is especially important when used for tissue engineering or other applications that require high strength, enhanced compressive and tensile properties, good elasticity, and endurance (for example, cartilage tissue) [101]. Furthermore, they are difficult to handle and use for specific applications because of their poor mechanical properties. Hydrogels research has recently shifted to optimize their chemical and mechanical properties, particularly for biomedical applications [102,103,104].

New varieties of hydrogels, including nanocomposite hydrogels, are being developed to improve the material properties of hydrogels and expand the range of their applications in medical and biotechnological fields. In addition, nanoparticles are also being applied in the consumer market. Despite their wide applications, the safety in the use of nanoparticles remains a significant challenge [105]. This obstacle can be overcome by combining them with hydrogels, resulting in lower environmental and human health risks. The hybrid combination of hydrogels and nanoparticles results in structurally diverse materials and improves their combined properties [106]. The large intermolecular spaces in the hydrogel networks serve as homes for a large number of nanoparticles and as nanoreactors for their synthesis [107]. Polymeric hydrogels act as a “host” that can accommodate various types of nanoparticles as a “guest” to form nanocomposite hydrogels [108].

The invention of nanoparticles into the hydrogel network results in the development of new materials with physical and biomedical properties that have promising applications in the biomedical field [109]. Thus, many nanocomposite hydrogels have been engineered for several biomedical applications, including drug delivery [110,111], biosensors [112], tissue engineering [113], and wound healing [114]. Past research has successfully developed an improved skin delivery of voriconazole with a nanostructured lipid carrier-based hydrogel formulation. The lipid nanoparticles were uniformly dispersed in the gel base, retaining their spherical shape and narrow distribution. All in all, these findings demonstrated that the NLC were homogeneously incorporated into the hydrogel while maintaining the beneficial properties of NLC dispersion, particularly small particle size and homogeneity [115].

### 4.3. Efficacy and Safety of NLC-Hydrogel

In order to understand the efficacy and safety of NLC-hydrogel, preclinical and clinical trials need to be carried out so that desirable results can achieve for future use.

#### 4.3.1. In Vitro Study

In vitro drug release study carried out by Tan G. et al. (2017) suggested that the dexamethasone (DXM)-NLC based hydrogel gave a sustained drug release with 88.65% of total DXM within 72 h. In contrast, DXM-NLC gave a fast drug released with 93.10% within 48 h. This showed that the DXM-NLC based hydrogel could release drug sustainably and may represent effective carriers for ocular sustained release delivery. The obtained results indicated that with regard to DXM-NLC based hydrogel, the drug has to undergo an additional barrier as a result of entrapment in NLC. This causes the release of DXM from NLC based hydrogel to retard accordingly giving sustained release of DXM over a long period of time [116]. A slow-release drug delivers consistent amounts of drug over a long period, and there is no need for the patient to take medication frequently. In addition, the same nanohybrid system was used for ocular drug delivery that showed the quercetin (QN)-NLC based hydrogel not only facilitates the transcorneal penetration of QN but also prolongs the precorneal retention time, supplying an assortment of favorable features that are not available with the isolated drug delivery system. The release rate of the drug from the QN-NLC based hydrogel was evidently lower than that from QN eye drops. The cumulative amount of released QN from QN eye drops was 99.38% within 12 h, and that QN-NLC based hydrogel was 80.55% within 72 h [117].

Furthermore, the nanohybrid system of the voriconazole (VR)-NLC based hydrogel formulation displayed retarded drug release where NLC dispersion showed a 95% release of VRC, whereas the NLC-gel released up to 76% of VRC within 24 h. The profile indicated that embedding NLC into hydrogel effectively controlled the release of VRC because the release of VRC from the NLC based hydrogel was a combination of the release of the drug from lipid carriers and subsequent diffusion through the micro-channel structures of the carbopol gel. In a clinical setting, this sustained release by hydrogel would provide the drug over a prolonged period of time [115]. Interestingly, the toxicity test of clotrimazole (CTZ)-NLC based hydrogel performed on HeLa cells showed low toxicity value after 24 h resulting in a cell viability of 77.2% compared to CTZ-NLC with only 24% cell viability [118]. Other studies found that the use of mucoadhesive and thermosensitive hydrogels composed of poloxamers (i.e., pluronic^®^ F127 and F68) and polycarbophil significantly reduced the toxicity of CTZ. The concentrations of CTZ showing 50% of cell viability were 53 and 70 μg/mL for CTZ in PBS and in the hydrogel [119].

#### 4.3.2. In Vivo Study

The development of tetracaine (TTC)-NLC based hydrogel displayed successful anti-nociceptive properties suitable for topical drug delivery in dentistry. The in vivo antinociceptive assay using mice demonstrated that TTC-NLC based hydrogel demonstrated a longer duration of analgesia (34 h) compared to TTC-hydrogel (8 h). Both the concentration of TTC and its encapsulation in NLC influenced the duration of analgesia. The lipid composition of the NLC may have favored storage of TTC at the site of administration, consequently prolonging the anesthetic effect on the mice. In addition, the moisture present in the mucus-covered nasal surface favors the process of mucoadhesion of TTC-NLC based hydrogel which supports the rapid onset action of drugs compared to the drier skin surface. This research opens up perspectives for the excellent anesthetic performance of TTC-NLC based hydrogel in humans, given that previous studies have observed that the flux of an anesthetic can be used to predict the efficacy of an anesthetic in the preclinical phase [120]. In addition, research on in vivo drug retention in epidermis and dermis of mice using 5-fluorouracil (5-FU)-NLC based hydrogel showed (91.256 ± 4.56 μg/cm^2^) as compared with that from the 5-FU hydrogel (12.23 ± 3.86 μg/cm^2^) in the rat skin.

The in vivo skin irritation was also significantly reduced the (5-FU)-NLC based hydrogel compared with 5-FU hydrogel. These outcomes show that the prepared nanohybrid system improves the penetration of 5-FU through the stratum corneum with minimal skin irritation, which is prerequisite for topically applied formulations [121]. Formulation of resveratrol (RES)-NLC based hydrogel was evaluated by Rajput et al. (2018) using an in vivo pharmacodynamic study by the scopolamine-induced amnesia model in rats using Morris Water Maze test for Alzheimer’s disease (AD). The Morris Water Maze model is very useful for spatial memory study. It is based on cues to locate a submerged escape platform from different starting points. Spatial learning was determined by conducting repetitive trials, and reference memory was determined by ability to find a platform area when the platform was absent [122].

The experiment showed that the rats in the normal control group (without treatment) showed shorter escape latency followed by RES-NLC based hydrogel and RES hydrogel. This implies that the developed formulation is more effective than RES hydrogel to treat AD. Moreover, the distance traveled by rats showed no significant difference between the normal control group and the RES-NLC based hydrogel group. The rats treated with the RES-NLC based hydrogel had better memory and could spend more time in a target quadrant compared to the normal control group. However, the rats treated with RES hydrogel had spent comparatively less time. The rats of the normal control group remembered and crossed the centrally placed platform 11 times in 90 s, which was similar to the RES-NLC based hydrogel treated group. However, the rats of the RES hydrogel treated group crossed the place of the platform only half of the time. Therefore, the RES-NLC based hydrogel formulation has potential to treat AD [123].

#### 4.3.3. Clinical Trials

Researchers can begin a human clinical trial after the design has been validated and individuals have been recruited. Study designs vary based on the researchers’ goals and may necessitate varying levels of participation from study participants. Participants in observational studies may be seen and tracked but not treated in order for researchers to discover how the condition progresses.

Tichota et al. (2014) planned a single-blinded, controlled trial for 30 days including ten volunteers for skin hydration study. The clinical trials were performed using an argan oil (AO)-NLC based hydrogel and an AO hydrogel (AO-HG) for topical delivery. According to the results, the AO-NLC based hydrogel formulation produced a significant increase in the skin hydration (*p* < 0.05) when compared to the AO-HG application. The significant increase in skin hydration observed for the AO-NLC based hydrogel formulation could be attributed to the presence of lipid nanoparticles which have displayed a skin-moisturizing effect when incorporated in the semisolid formulations. In addition, this effect could also be attributed to the argan oil, which has been described as a compound with skin-moisturizing properties [124]. Moreover, a prospective double blinded randomized controlled study was performed on 27 patients with neuropathic diabetic foot ulceration (DFU) for 8 weeks.

The study intended to investigate the wound healing of DFU using phenytoin (PHT), PHT- NLC based hydrogel and blank hydrogel. The results claimed that the ulcer size was significantly reduced with 95.82 ± 2.22% for PHT-NLC-hydrogel in comparison to 47.10 ± 4.23% and −34.91 ± 28.33% for PHT and blank-hydrogel, respectively. The patients using PHT-NLC based hydrogel were less exudative when compared to the negative control group. This may be attributed to the antibacterial activity of phenytoin when applied topically, which may have resulted in a reduction of the bacterial load within the wound bed. In short, PHT-NLC hydrogel is more effective in wound closure when compared to the positive and negative controls throughout the duration of the study [125]. Another study on anti-acne activity, skin elasticity, and hydration was performed by clinical examination after the use of a marigold extract (ME)-NLC based hydrogel. The study was performed on human subjects, under dermatological control for 28 consecutive days based on comedolytic and sebum regulating effects.

At the end of the examination, there was a significant decrease in the sebum rate with an average value of 63.95 μg/cm^2^ and a significant increase in the hydration level with an average value of 10.99 units. A drastic remission of the number of non-inflammatory retention lesions (elemental acne lesions) and inflammatory lesions (papules, pustules, and nodules) was determined. These results support ME-NLC based hydrogel to have non-comedogenic and non-acnegenic potential. Furthermore, the degree of the skin hydration effect was determined to be up to 74%, and skin elasticity reached 90% [126]. The high degree of hydration and elasticity demonstrated that topical application of the ME-NLC based hydrogel creates a monolayer lipid film on the skin, leading to an occlusive effect, and hence, it prevents transepidermal water loss (TEWL). This increases the skin hydration significantly [11,16]. Additionally, the occlusive effect can promote the deposition of drugs into the viable skin by reducing corneocytes packing and widening inter-corneocytes gaps [127]. Similarly, the skin hydration study of the vitamin E-NLC based hydrogel observed a significantly improved reduction in the skin TEWL (*p* < 0.05). This occurrence might be related to the formation of an occlusive film on the skin surface, which prevents the water loss by evaporation [128].

### 4.4. Application of Nanohybrid System: Nanostructured Lipid Carrier-Hydrogel in Drug Delivery

Overall, the advantages of the nanohybrid system containing the combination of two different materials (NLC and hydrogels) result in innovative materials with unique properties not found in the individual components, which are valuable in the drug delivery system. In one study carried out by Azar et al. (2021), a formulation of an olive leaf extract-NLC based hydrogel is a suitable carrier for olive leaf extract (OLE) to maintain its antioxidant properties against environmental factors and also could be considered in high-fat foods to reduce oxidation and enhance their nutritional properties. The formulation was carried out using the hot-high shear homogenization method, and then, the OLE-NLC was loaded in sodium caseinat–pectin hydrogel. Based on the study, the encapsulation efficiency of the OLE-NLC based hydrogel was 80%, which indicates the partial release of the OLE due to stirring and also its partial loss due to heat during production [46]. Another work by Ravani et al. (2013) developed a new formulation of a clotrimazole (CTZ)-NLC based hydrogel that could be proposed as an innovative system to administer CTZ to treat vaginal infection with *C. albicans.* The formulation was prepared using the ultrasonication method and viscosized by the addition of poloxamer P407 in the NLC dispersion (CTZ-NLC based hydrogel). These systems exhibit well-known thermogelling properties. The formulation showed a sol–gel transition value lower than body temperature and even after the addition of simulated vaginal fluid (SVF), the hydrogel maintained typical thermosensitive behavior [118]. A skin depigmenting agent was formulated from a Passiflora edulis seeds oil-(PESO)-NLC based hydrogel NLC. The formulation was prepared by using the ultrasonication technique and glyceryl distearate as a solid lipid and then gelled with Poly(acrylic acid) to form PESO-NLC based hydrogel. From the study, tyrosinase inhibitory activity and skin retention of the nanoparticles was superior to that of the non-encapsulated PESO. The developed formulations did not show cytotoxicity towards HaCat cells and presented suitable viscosity and texture properties for skin application, proving to be good candidates as depigmenting agent [129]. Table 3 summarizes recent nanohybrid systems (NLC-hydrogel) and their function in drug delivery.

## 5. Conclusions and Outlook

Substantial progress has been achieved in improving the properties of nanohybrid systems for drug delivery that use nanostructured-lipid-carriers-based hydrogels, as well as expanding the range of drugs and kinetics that can be achieved using this nanohybrid-based delivery vehicle. Even so, several questions remain to be answered to improve the clinical applicability of this nanohybrid drug delivery system. Issues such as the relationship between preclinical trials on animals and clinical modelling on humans and the nano-toxicity of different materials for different treatments may arise in the future. In the years ahead, nanostructured-lipid carriers-based hydrogel hybrid system designs will not only contribute to advanced applications but will also be a guide through fundamental knowledge of material interactions, allowing for computational prediction (e.g., molecular dynamic simulation) in drug delivery.

## Figures and Tables

**Table 1:** Preparation method of NLC with its advantages and limitations.

Method	Advantages	Limitations	References
High pressure homogenization (HPH)	A well-known and widely used technique. It is a simple and low-cost technique. Product with a more homogeneous particle size distribution and better overall stability. Both aqueous and non-aqueous dispersion media are employed.	It is not possible to completely avoid drug exposure to high temperatures. Incompatible with thermolabile drugs.	[12,13,14,19]
Solvent emulsification-evaporation	Large-scale production is feasible.	Uses organic solvent	[15]
Phase inversion	It is related to the two procedure. The inversion procedure needs three temperature cycles (85–60–85 °C).	Cumbersome technique	[16]
High speed homogenization and/or ultrasonication	Low particle size: 30–180 nm Low shear stress	Metal shading leads to contamination Energy intensive process	[17]
Solvent injection/displacement	Easy handling and fast production process Lipids are dissolved in water missicible solvent	Use organic solvent	[18]

**Table 3:** Summary of recent nanohybrid system a function in drug deliver.

Nanohybrid Drug Delivery System	Active Ingredient/Drug	Function	Particle Size	References
Olive leaf extract-NLC based hydrogel	Olive leaf	Antioxidant	303 nm	[46]
Baicalin-NLC based hydrogel	Baicalin	Anti-inflammatory in ocular drug delivery	99.64 nm	[130]
Whey protein-NLC based hydrogel	Whey protein	Oral drug delivery	347 nm	[131]
Clotrimazole-NLC based hydrogels	Clotrimazole	Anti-fungal	-	[118]
Tea tree oil-NLC based hydrogel	Tea tree oil	Wound healing	-	[132]
Ascorbyl palmitate-NLC based hydrogel	Ascorbyl palmitate	Skin moisture	268 nm	[133]
Valdecoxib-NLC based hydrogel	Valdecoxib	Anti-inflammatory	170 nm	[134]
Dexamethasone-NLC based hydrogel	Dexamethasone	Ocular delivery system	-	[117]
Voriconazole-NLC based hydrogel	Voriconazole	Antifungal	212.2 nm	[115]
Passiflora edulis seeds oil-NLC based hydrogel	Passiflora edulis seeds oil	Skin depigmenting agent	150 nm	[129]

## Data Availability

Not applicable.
